# The Use of Virtual Reality in Craving Assessment and Cue-Exposure Therapy in Substance Use Disorders

**DOI:** 10.3389/fnhum.2014.00844

**Published:** 2014-10-17

**Authors:** Antoine Hone-Blanchet, Tobias Wensing, Shirley Fecteau

**Affiliations:** ^1^Laboratory of Canada Research Chair in Cognitive Neuroscience, Centre Interdisciplinaire de Recherche en Réadaptation et Intégration Sociale, Centre de Recherche l’Institut Universitaire en Santé Mentale de Québec, Faculté de Médecine, Université Laval, Quebec, QC, Canada; ^2^Berenson-Allen Center for Non-invasive Brain Stimulation, Beth Israel Deaconess Medical Center, Harvard Medical School, Boston, MA, USA

**Keywords:** substance use disorders, craving, virtual reality, avatars, cue exposure

## Abstract

Craving is recognized as an important diagnosis criterion for substance use disorders (SUDs) and a predictive factor of relapse. Various methods to study craving exist; however, suppressing craving to successfully promote abstinence remains an unmet clinical need in SUDs. One reason is that social and environmental contexts recalling drug and alcohol consumption in the everyday life of patients suffering from SUDs often initiate craving and provoke relapse. Current behavioral therapies for SUDs use the cue-exposure approach to suppress salience of social and environmental contexts that may induce craving. They facilitate learning and cognitive reinforcement of new behavior and entrain craving suppression in the presence of cues related to drug and alcohol consumption. Unfortunately, craving often overweighs behavioral training especially in real social and environmental contexts with peer pressure encouraging the use of substance, such as parties and bars. In this perspective, virtual reality (VR) is gaining interest in the development of cue-reactivity paradigms and practices new skills in treatment. VR enhances ecological validity of traditional craving-induction measurement. In this review, we discuss results from (1) studies using VR and alternative virtual agents in the induction of craving and (2) studies combining cue-exposure therapy with VR in the promotion of abstinence from drugs and alcohol use. They used virtual environments, displaying alcohol and drugs to SUD patients. Moreover, some environments included avatars. Hence, some studies have focused on the social interactions that are associated with drug-seeking behaviors and peer pressure. Findings indicate that VR can successfully increase craving. Studies combining cue–exposure therapy with virtual environment, however, reported mitigated success so far.

## Introduction

Substance use disorders (SUDs), commonly referred to as drug addictions, are a public health issue of growing importance throughout the world. Indeed, the number of individuals diagnosed with SUDs exceeds 15 million worldwide (World Health Organization, 2014), leading to annual costs of more than $600 billion in the United States only (National Institute on Drug Abuse, 2014). Several legal and illegal substances entail psychoactive characteristics that may interfere with the normal functioning of individuals, providing pleasure, and chronic intake of these substances can induce SUDs. Although the majority of these active compounds are well known from pharmacological and toxicological viewpoints, prevention of relapses and treatment of SUDs remain difficult as psycho- and pharmacotherapies offer relatively poor success rates. Combination of medication and behavioral assessment is becoming increasingly important in addiction medicine and more attention is dedicated to find ways to improve such combinations. Virtual reality (VR) and novel computer technologies are well established in the fields of neurosciences and psychology and may prove to serve as supplementary tools in the assessment and treatment of SUDs. In this review, we summarize current knowledge on craving assessment and cue-induced therapy in SUDs using VR technologies.

Clinically defined as a chronically relapsing disorder, the DSM 5 criteria for SUDs include the escalation of substance intake, appearance of tolerance, difficulties in limiting the intake, emergence of serious withdrawal symptoms and negative affect during limiting the intake, drug seeking, and craving (DSM 5, 2013). Psychoactive substances, from nicotine and alcohol to crack cocaine and methamphetamine (METH), all have differential effects on the brain and induce different psychophysiological consequences. However, all these compounds directly or indirectly stimulate the dopamine neural pathways, commonly referred to as the reward pathways, providing pleasure through intoxication. Subjective pleasure associated with recreational substance use and physiological habituation to the substance may then motivate subsequent intake and trigger an addictive process, which requires more effort and time to obtain and use the substance. Chronic substance use is, therefore, characterized by escalation of intake and loss of control in limiting the intake, emergence of withdrawal symptoms and negative emotional states when limiting the intake, and compulsive drug-seeking behavior (Koob and Volkow, 2009). Importantly, drug-seeking behavior and the negative affect associated with withdrawal are critical in relapse.

### Craving is a critical factor in SUDs

Craving is defined in the DSM 5 as the intense preoccupation or urge to use the desired substance, is a complex phenomenon, encompassing neurobiological and psychological mechanisms. The DSM 5 added craving as a crucial diagnosis criterion of SUD. Although this remains disputed, craving is also considered a predictive factor of relapse (Paliwal et al., 2008; Galloway and Singleton, 2009).

From a neurocognitive perspective, addiction is seen as an unbalance between reflexive and reflective systems (Bechara, 2005). Reflective processes, on the one hand, encompass cognitive inhibitory control and delayed discounting of available reward, an ability to resist craving and make adapted decisions. These processes depend on cerebral structures associated with executive functioning, such as the dorsolateral (DLPFC) and orbitofrontal (OFC) subdivisions of the pre-frontal cortex (PFC). On the other hand, reflexive processes are related to impulsivity and risky decision-making, the motivational and emotional responses to reward, which highly depend on activity in the basal ganglia and limbic areas. In fact, cognitive downregulation of craving is associated with increased activity of the PFC and decreased activity in the nucleus accumbens, ventral tegmental area, and amygdala (Brody et al., 2007; Kober and Mende-Siedlecki, 2010). In patients with SUDs, impairments in reflective and reflexive processes are believed to facilitate a disregard toward adaptive decision-making and facilitate compulsive drug seeking.

From a neurophysiological perspective, craving is initiated when the reward pathways, habituated with frequent and intense firing of dopamine neurons from the mesocorticolimbic projections to the nucleus accumbens and DLPFC, are kept relatively understimulated for a certain period of time, as when one remains abstinent. Several functional magnetic resonance imaging (fMRI) studies have demonstrated that abstinence-induced craving increases cerebral blood flow and neuronal activity in the DLPFC, nucleus accumbens, and limbic structures including the hippocampus and amygdala (Tomasi et al., 2007; Wang et al., 2007).

Thus, it is generally accepted that addiction is *initiated* by chronic and repetitive overstimulation of the dopamine pathways, but its *maintenance* is highly dependent on more complex cerebral mechanisms, such as emotional regulation and memory. These processes involve several cerebral structures other than the basal ganglia including the PFC, basolateral amygdala, and hippocampus (Steketee and Kalivas, 2011; Barak et al., 2013). Memories and learned behaviors toward an artificial reward are thus reinforced during periods of drug use in comparison to natural rewards, creating perturbations in inhibitory control and reflective processes.

### Socioenvironmental factors are important in craving

Individuals with a diagnosis of SUD, including those in state of recuperation, and abstinent individuals are particularly vulnerable to social and environmental cues related to substance abuse. Specific drug-related environments (e.g., pubs and nightclubs) and social interactions (e.g., peer pressure) can provoke craving and relapse in abstinent individuals (Ferguson and Shiffman, 2009). Indeed, this sensitivity to drug-related conditioned cues and anticipation of future drug reward is thought to provoke a potentiated neural response in the neural pathways of reward. fMRI studies have demonstrated that cue-induced craving, for different substances of abuse, increases activity in the amygdala, anterior cingulate cortex (ACC), and PFC (Schneider et al., 2001; Seo et al., 2013). Of utmost importance, limbic activation and inception of craving following the presentation of drug cues are relatively independent of a pharmacological abstinence state and may also be elicited after drug administration (Franklin et al., 2007). Moreover, abstinence and withdrawal states may potentiate cue-induced craving in the PFC and striatum (McClernon et al., 2008). Studies using positron emission tomography (PET) imaging, which allows the imaging of a radioactive compound acting as a competitive agonist to certain receptors, have shown that cocaine- and nicotine-related cues induce changes in metabolic activity in the striatum, ACC, and different regions of the PFC (Brody et al., 2002; Volkow et al., 2007). These results demonstrate that SUD is a pathology of neuroplasticity and that drug-associated socioenvironmental cues provoke behavioral (the sensation of craving) and neurobiological (activity patterns in sensitive brain areas) responses.

Hence, as craving is now considered a crucial factor in the process of SUDs, its assessment is essential for future identification, management, and treatment outcomes. Craving episodes may vary in intensity, latency, frequency, and salience, and are traditionally measured with craving questionnaires and visual analog scales (VAS), in which individuals report subjective states of craving ranging from “not at all” to “very much” (cf., Bordnick et al., 2005; Culbertson et al., 2010). The assessment of craving along the course of therapeutic modalities is thought to represent a good marker of rehabilitation and treatment success, although this idea is still disputed (Perkins, 2012), and the majority of current studies in SUDs reports craving scores as primary or secondary outcomes. Current ways of inducing craving rely mostly on visual cues, such as pictures or videos presenting cigarettes and drugs, or the combination of such visual cues with other sensory modalities (e.g., the smell of a cigarette). Although these cues are relevant to drug-seeking behavior and successfully induce craving, they do not trigger responses associated to social or environmental cues *per se* (i.e., a specific locale or social interaction). VR paradigms may represent an improvement in this regard, as it enables the reconstruction of specific environments and characters (i.e., a bar and a bartender) and allows a certain social immersion and interaction within this context (Kuntze et al., 2001; Lee et al., 2003).

### Cue-exposure therapy has therapeutic potential in craving reduction

Studies now support the idea that intervention on sensitivity and reactivity to environmental context and cues is necessary to shoulder pharmacotherapy in the treatment of SUDs (Perry et al., 2011). Cue-exposure therapy (CET) as an intervention has been used extensively in the past with patients suffering from social phobias and agoraphobia (Vögele et al., 2010), fear of flying (Maltby et al., 2002; da Costa et al., 2008), post-traumatic stress disorder (Reger and Gahm, 2008), and generalized anxiety disorders (Hofmann et al., 2006).

Cue-exposure therapy is primarily based on Pavlov’s concept of classical conditioning, a main theory of learning in behavioral science. In classical conditioning, an unconditioned cue or stimulus (UCS) evoking an unconditioned response (UCR) is paired with a neutral cue for a longer period of time until the neutral stimulus itself can result in a response similar to the UCS. This response is referred to as a conditioned response (CR), which is elicited by the no longer neutral, but conditioned stimulus (CS). Thus, the concept of classical conditioning has been demonstrated to be a key mechanism of a variety of psychological phenomena, including persistent substance use and abuse (Drummond et al., 1990). The drug serves as the UCS, which elicits the UCR, usually an automatic physiological response (e.g., craving). Pairing the UCS with a neutral cue (e.g., an object such as a syringe or an environment such as a restaurant) results in a CR that evoked by the mere presence of the CS and independent of the substance itself. Hence, constant substance abuse leads to increases in cue reactivity by establishing an association between the cue and the physiological response. CET aims at diminishing the conditioned relation between a substance-related cue and the physiological response by systematically pairing them in a treatment setting. The constant combination of a CS with a CR in absence of the actual drug reduces the physiological reactivity to a substance-related cue, since no drug is actually induced. This eventually results in an extinction of the cue-response association and, therefore, decreases reactivity to substance-related cues, which might be held responsible for maintained levels of craving.

Through the currently available results, success of CET in treating SUDs is mitigated. A meta-analysis conducted in 2002 (Conklin and Tiffanny, 2002) concluded that CET is not significantly more efficient than any other behavioral cognitive therapy, although the authors state that the limited amount of results did not allow a clear interpretation of the phenomenon. Of great interest, Martin et al. (2010) have recently reviewed several studies with different methodologies (some of them combining VR paradigms, pharmacological augmentation, and fMRI with CET) in different SUDs (nicotine, alcohol, cocaine, and opiates). Although only four of the reviewed studies were randomized clinical trials, the authors conclude that CET demonstrates interesting therapeutic potential, although not significantly superior to other available treatments (Martin et al., 2010). Importantly, they also mention that several methodological issues in CET studies are to be addressed in the future to warrant significant benefits.

In the following sections, we will discuss results on (i) the use of VR in assessing cravings in SUDs and (ii) the combination of VR and CET to tune down craving intensity and remediate to substance use in SUDs. Furthermore, this review separates findings of studies using VR to recreate computer-generated environments as opposed to others proposing social interactions with avatars in addition to virtual environments, both in the context of SUDs.

## Data Review

### Virtual reality as a supplementary tool for craving assessment

Virtual reality enables the simulation of drug-related cues and environments to induce craving, and thus might serve as an ecologically valid addition to traditional craving assessments. While virtual environments by themselves – as well as possible interactions with avatars in another virtual environment – may induce craving, their specific nature of induction differs within and across VR paradigms. Therefore, we have separated studies proposing only the exposure to VR environments and studies using both VR environments and the possibility of social interaction with avatars. Tables 1 and 2 provide summaries of each study’s design and results.

**Table 1:** **Summary of studies assessing craving induction by substance-related cues in VR settings using virtual environments**.

Author (Year)	*N*^a^	Type of patient^b^	Hardware^c^	Craving assessment (scale)^d^	Effects of VR environments on subjective craving^e^	Effects of VR environments on subjective craving^f^
**ALCOHOL**						
Ryan et al. (2010)	23	Binge Drinkers	HMD	1-item Likert (0–10)	Subjective craving was higher for binge drinkers compared to controls	
**METHAMPHETAMINE**						
Culbertson et al. (2010)	17	METH users	Screen	9-item VAS (0–100)	VR increased craving for cannabis related compared to neutral cues	Increases in LF HR-power were associated with high craving, those in HF HR-power with low craving
**NICOTINE**						
Acker and MacKillop (2013)	47	Moderate ND^1^	HMD	5-item VAS (0–100)	VR increased craving for smoking related compared to neutral cues	
Baumann and Sayette (2006)	20	Moderate ND^1^	Screen	1-item VAS (0–100)	VR increased craving for smoking related compared to neutral cues	
Gamito et al. (2011)	32	Students	Screen	10-item QSU (1–7)	VR increased craving for smoking related compared to neutral cues only in smokers and not in non-smokers	
Lee et al. (2003)	21	Moderate ND^1^	HMD	1-item VAS (0–100)	VR led to an increase in craving, whereas pictures did not	
Lee et al. (2005)	8	Moderate ND^1^	HMD	1-item Likert (1–10)	Craving increased from pre- to post- fMRI scanning sessions	Smoking-related VR cues increased activity in PFC, STG, cerebellum compared to neutral cues
Paris et al. (2011)	24	Daily smokers	HMD	1-item Likert (1–10)	VR increased craving for smoking related compared to neutral cues. VR increased craving for context-related, but non-smoking, cues compared to neutral cues	
Pericot-Valverde et al. (2011)	46	Students^2^	HMD	1-item VAS (0–100)	Both VR environments significantly induced craving.	
Traylor et al. (2009)	20	ND^3^	HMD	3-item ACVAS (0–10)	VR increased craving for smoking related compared to neutral cues	
Traylor et al. (2009)	14	ND^3^	Screen	VAS	VR increased craving for alcohol-related cues in the office and a neutral scene in nicotine-dependent/alcohol-consuming individuals, but not nicotine-dependent/non-alcoholconsuming individuals. VR increased craving for nicotine-related cues in the smoking-related scenes, but not the initially presented neutral scene for both groups	

*VR, virtual reality*.

*^a^Number of participants assigned to experimental group of substance users*.

*^b^Clinical or diagnostic characteristics of substance users according to the study’s authors, ND, nicotine dependence [^1^Meeting criteria for nicotine dependence and severity of dependence based on the Fagerstrom Test of Nicotine Dependence (FTND), ^2^Minimum smoking rate of 10 cigarettes per day, ^3^Meeting DSM-IV-TR criteria for nicotine dependence]*.

*^c^HMD, head mounted display; screen, computer screen placed in front of the participant*.

*^d^Tool of assessment for subjectively reported craving levels before, during and after cue exposure, Likert, Likert Scale; VAS: Visual Analog Scale; QSU, Questionnaire of Smoking Urges; ACVAS, Attention to Cues Visual Analog Scale*.

*^e^fMRI, functional magnetic resonance imaging*.

*^f^LF/HF, low frequency/high frequency; HR, heart rate; PFC, pre-frontal cortex; STG, superior temporal gyrus*.

**Table 2:** **Summary of studies assessing craving induction by substance-related cues in VR settings using virtual social interactions**.

Author (Year)	*N*^a^	Type of patient^b^	Hardware^c^	Craving assessment (scale)^d^	Effects of VR social interactions on subjective craving	Effects of VR social interactions on physiological correlates of craving^e^
**ALCOHOL**						
Bordnick et al. (2008)	40	AUD^1^	HMD	1-item VAS (0–100)	VR increased craving for alcohol related compared to neutral cues; Aversive cues (argument) elicited less craving than the other alcohol-related cues	
Cho et al. (2008)	10	AD^2^	Screen	1-item VAS (0–100)	Subjective craving was higher for cues with an avatar providing social pressure than without, regardless of the presence of alcohol; subjective craving differed in presence of alcohol cues and absence of the avatar, but not vice versa	
Lee et al. (2008)	14	AD^3^	HMD	1-item VAS (0–100)	Subjective craving in alcohol-dependent subjects was mainly induced by alcohol-related cues and did not differ w.r.t. presence/absence of social pressure; social pressure increased alcohol craving for control subjects, who did not show craving to alcohol-related cues	Increase in EEG alpha-power at right frontal sites
**CANNABIS**						
Bordnick et al. (2009)	20	CUD^4^	HMD	1-item CCVAS (0–100)	VR increased craving for METH related compared to neutral cues	
**COCAINE**						
Saladin et al. (2006)	11	CCD^5^	HMD	1-item VAS (0–100)	VR increased craving for cocaine related compared to neutral cues	HR increased for some cocaine-related cues, but did not reveal single differences between cues
**NICOTINE**						
Bordnick et al. (2004)	13	ND	HMD	1-item VAS (0–100)	VR increased craving for smoking related compared to neutral cues	
Bordnick et al. (2005)	10	ND	HMD	1-item VAS (0–100)	VR increased craving for smoking related compared to neutral cues	SC increased during VR smoking-related, but not neutral cues
Carter et al. (2008)	22	ND^6^	HMD	24-item MDS (0–11)	VR increased craving for smoking related compared to neutral cues	
Ferrer-García et al. (2010)	25	Students^7^	HMD	1-item VAS (0–100)	All virtual environments induced significant craving, in high- or low-dependent subjects	
Garcìa-Rodrìguez et al. (2012)	46	Daily smokers^7^	HMD	1-item VAS (0–100)	VR increased craving for smoking related compared to neutral cues	HR and T increased during some but not all smoking related VR cues compared to neutral cues; SC did not differ across conditions
Garcìa-Rodrìguez et al. (2013)	45	Daily smokers^7^	HMD	1-item VAS (0–100)	Smoking a virtual cigarette increased craving compared to neutral cues	Smoking a virtual cigarette increased HR compared to neutral cues

*VR, virtual reality*.

*^a^Number of participants assigned to experimental group of substance users*.

*^b^Clinical or diagnostic characteristics of substance users according to the study’s authors, AUD, alcohol use disorder; AD, alcohol dependence; CCD, crack cocaine dependence; ND, nicotine dependence [^1^Meeting DSM-IV-TR criteria for alcohol abuse or dependence, ^2^Based on the Alcohol Dependence Scale (ADS), ^3^Abstinence for at least 3 weeks, ^4^Meeting DSM-IV-TR criteria for cannabis abuse or dependence, ^5^Meeting DSM-IV criteria for crack cocaine dependence, ^6^Meeting DSM-IV-TR criteria for nicotine dependence, ^7^Minimum smoking rate of 10 cigarettes per day]*.

*^c^HMD, head mounted display; screen, computer screen placed in front of the participant*.

*^d^Tool of assessment for subjectively reported craving levels before, during, and after cue exposure, AAS, Alcohol Attention Scale; VAS, visual analog scale; CCVAS, Cannabis Craving Visual Analog Scale; MDS, Multidimensional Scaling*.

*^e^EEG, electroencephalogram; HR, heart rate; SC, skin conductance; T, body temperature*.

#### VR environments inducing drug cravings

Kuntze et al. (2001) developed the first pilot study combining a classical cue-exposure paradigm with VR in heroin dependent males. Their methodological design specifically compared craving ratings following counterbalanced cue exposure to an immersive virtual environment, cue exposure to pictures, and to neutral stimuli. Cravings were measured with a VAS and the Yale–Brown Obsessive Compulsive Scale (YBOCS) on VAS before, during, and after exposure to the different sets of cues. Several physiological measurements were also obtained, including electroencephalography (EEG), skin conductance, and cardiac rhythm. Although the authors did not expound on the actual results of the study, their methodological development paved the way for other works (Kuntze et al., 2001).

The following set of studies investigating the effect of VR environments in eliciting cravings largely focused on nicotine intake and smoking. Tobacco use disorder (TUD) (DSM-5) is still a leading cause of preventable deaths and illness throughout the world and represents a complex condition (Rose, 2005). Although several pharmacotherapies are available, success rates remain relatively disputable and total abstinence is hard to achieve. Lee et al. (2003, 2005) pioneered the works on VR in TUD with two landmark studies. In their pilot study, they sought to determine if VR environments evoked nicotine cravings more effectively than presenting normal two-dimensional cues, such as photographs (Lee et al., 2003). They designed a VR bar, which had previously been identified as an environment eliciting superior cravings in smokers from results of a questionnaire. The 22 subjects participating in the study were randomly assigned to a group exposed to either VR or photographs and exposed to this condition for 5 min. Craving ratings were collected with a single item on VAS inquiring on the current urge to smoke. The VR bar induced significantly greater nicotine cravings than the photograph condition. This supports the idea initiated in Kuntze’s pilot study that exposure to VR smoking-related cues results in elevated craving ratings when compared to classical cue-exposure designs. Following these results, Lee et al. (2005) presented the same task during fMRI acquisition to determine neural correlates of VR cue reactivity (Lee et al., 2004). The eight subjects participating in the study were required to complete a craving VAS before and after the scan, during which they experienced immersion in a VR bar and exposure to two-dimensional and three-dimensional smoking-related and neutral cues through MR-compatible goggles. fMRI scans allowed to measure a significant increase in brain activity in the PFC, ACC, inferior temporal cortex, and supplementary motor area following the presentation of two-dimensional smoking versus neutral cues, consistently with neuroimaging data on craving. Interestingly, following the immersion in the VR bar, significant brain activation was only recorded in the PFC and cerebellum. These results demonstrate that 3D stimuli in a cue-provoked paradigm may be as efficient as 2D stimuli, since they induce a response in the PFC, which has been associated with craving. Activation in the cerebellum may be related to the control of posture and balance, required in the 3D environment. The authors also identified a limitation in their recent VR bar paradigm; the smoking cues were scattered around the environment and may have required additional attentional resources compared to the photographs. Works from Baumann and Sayette (2006), Traylor et al. (2009), and Pericot-Valverde et al. (2011) have also explored the efficacy of VR in nicotine craving inducement. In these studies, smokers were exposed to different virtual environments (neutral and smoking-related) and required to rate their craving levels. In Baumann and Sayette’s (2006) study, smoking deprived subjects rated their cravings significantly higher following exposure to the smoking-related environment (Baumann and Sayette, 2006). Interestingly, Traylor et al.’s (2009) study implemented olfactory stimuli to VR environments and tested this paradigm in non-abstinent smokers (Traylor et al., 2009). They found that smoking-related VR cues significantly increased cravings in smokers compared to neutral cues, even though the subjects had been allowed to smoke before the experiment. However, the olfactory stimuli condition did not affect craving levels. The last two studies have clearly demonstrated how VR smoking-related cues may successfully induce craving in smokers, independent of the state of abstinence. Pericot-Valverde et al. (2011) demonstrated the different evolution patterns of subjective craving for cigarettes in VR environments, with smoking-related environments provoking a more rapid increase in subjective craving intensity (Pericot-Valverde et al., 2011). Moreover, Gamito et al. (2011) examined the difference between three different VR environments in a study comparing smokers and non-smokers. Results were similar to previously discussed studies, as high arousal virtual settings provoked the greatest significant increases in subjective craving for cigarettes in smoking individuals. Smokers and non-smokers reported similar scores for sense of presence and cybersickness (Gamito et al., 2011). The aforementioned studies have well illustrated the efficiency of VR environments in inducing cravings, but also the importance of specificity and context in VR cues to facilitate a reaction. The role of context in craving has been directly assessed in a study by Paris et al. (2011), in which multiple smokers were exposed to four different VR environments including two neutral nature scenes and two identical convenience stores; one comprising the usual available tobacco and cigarettes and one stripped from all smoking-related products (Paris et al., 2011). Subjects spent 3 min in each environment in a counterbalanced randomized order and indicated their craving level on a 10-point scale. Cravings were significantly increased following exposure to the convenience store with smoking cues compared to neutral environments. More importantly, results also showed an increase in subjective cravings between the first nature scene and the convenience store *without* smoking cues. This provides additional evidence that a smoking-related scenery without explicit smoking cues may provoke craving (Conklin, 2006; Conklin et al., 2008). This finding may possibly be attributed to the subject’s own expectations (McBride et al., 2006), and reinforces the idea that VR is an adequate medium to study cravings for nicotine. Finally, Acker and MacKillop (2013) obtained similar results when comparing the effect of exposure to a neutral or smoking-related virtual environment on cravings in smokers, but also investigated cigarette craving from a behavioral economic perspective. They found that as craving levels increased following the exposure to a smoking-related virtual environment, the demand index (i.e., representing the economical cost subjects were willing to pay for a cigarette) also increased (Acker and MacKillop, 2013). This is in line with the literature on impaired decision-making, increased compulsive behavior and risk-taking in addicted individuals (Goldstein and Volkow, 2002; Lejuez et al., 2003; Garavan and Stout, 2005; Jentsch and Pennington, 2013).

Virtual reality has also been utilized to induce craving in alcohol use disorder (AUD), a complex relapsing disorder with current moderate therapeutic success and in need of novel intervention techniques (Addolorato et al., 2012). With alcohol being a widely available substance, differentiation of social drinkers and pathological users is critical (Myrick et al., 2004), as is the identification of concomitant substance use. Traylor et al. (2009) sought to determine if cravings for nicotine and alcohol differed in three VR environments (i.e., a neutral scene, a party scene, an office scene) in alcohol-dependent and non-alcohol-dependent smokers/daily drinkers. Results showed that alcohol-dependent subjects had significantly increased alcohol cravings in all three VR environments, whereas non-alcohol-dependent subjects only showed a significant increase in craving when exposed to the party scene. Interestingly, non-alcohol-dependent smokers were significantly more sensitive to smoking-related VR cues than the alcohol dependent, raising interesting questions on cross-sensitization and cue differentiation. The authors propose that alcohol cues may have provoked a ceiling effect in alcohol-dependent subjects since they were required to remain abstinent 24 h before the experiment. Although this may represent a limitation, the results demonstrate that VR paradigms can successfully induce cravings for alcohol. Ryan et al. (2010) also successfully induced alcohol cravings with VR cues in binge drinkers. Simultaneous to the immersion of four VR environments, subjects were exposed to olfactory (i.e., scents of whiskey and cigarettes) and auditory cues (Ryan et al., 2010). Binge drinkers compared to non-binge drinkers showed a significant increase in alcohol cravings in two out of four contexts, the party and the kitchen scenes, whereas both groups showed an increase in alcohol craving within the bar scene. Binge drinkers also reported significantly higher levels of thinking about drinking. Although it can be argued that binge drinkers do not necessarily fit the diagnosis criteria for AUD and that the control sample size was smaller, these results show that the desire to drink can be successfully induced with VR in regular drinkers, enabling to monitor early onset signs of SUD.

Apart from previous works on TUD and AUD, one study has been conducted to induce cravings for METH making use of VR environments. METH is a powerful psychostimulant with important addictive potential and dangerous neurological and physiological side effects (Marshall and O’Dell, 2012). In a VR study, non-treatment-seeking METH users were exposed to a VR neutral room and a METH-related room, in which drug transactions occurred, avatars used the drug and loud music played, and reported subjective cravings and emotional states on a VAS (Culbertson et al., 2010). The authors measured four aspects of craving, asking the subjects if they crave, desire, want, and would use METH right now. All aspects were measured on VAS. The cue condition significantly increased ratings for all four craving items and one anxiety item, when compared to the neutral VR room. Moreover, the VR cues induced greater cravings than METH-related videos, although this effect was not statistically significant. Again, this work contributes to demonstrate the viability and validity of VR in addiction research. Of technical interest, the study design was conducted with a free online VR platform, which advocates for the accessibility and relative logistical simplicity of VR in studying cue-provoked paradigms.

In summary, the studies discussed above unanimously show that immersion in VR environments can successfully induce cravings for a wide range of substances (see Table 1). Although works have primarily focused on TUD and AUD, several lines of evidence demonstrate that cue-induced craving with traditional stimuli can be induced with other substances (Carter and Tiffany, 2001) such as marijuana (Filbey et al., 2009), cocaine, and synthetic psychostimulants (Garavan et al., 2000; Bonson et al., 2002; Kühn and Gallinat, 2011). It can thus be suggested that future studies will support the efficacy of VR in inducing cravings in other SUDs. Although it can be disputed that self-reported measures may not always be as accurate as neurobiological correlates in assessing craving levels, it has to be acknowledged that most of the studies discussed above included important sample sizes and control conditions with either a comparison with healthy subjects or with a neutral VR condition or both. Future works will surely rely on simultaneous brain imaging to provide solid results.

#### VR and social interactions with avatars

Several studies assessing the use of VR to induce craving frequently expose subjects to environments depicting people, which perform substance-related behavior (i.e., talking about or administering a specific drug). These representations of human individuals in a digitalized form are usually referred to as *avatars*. The use of the term avatar itself is rather ill-defined and, thus, often used inconsistently across experimental studies. A general and broader use of an avatar in most studies includes all digital representations of a person in a virtual environment (Lim and Reeves, 2009). Experimental settings with this kind of avatar usually provide a VR scenario, such as a bar or a party, in which the subject is generally limited to the role of an observer. This approach allows a high degree of standardization, leaving the experimenter in charge of controlling the subjects’ field of view and gaze, and guiding them through a scenario in a specific order while exposing them to stimuli for a predefined period of time (Stoermer et al., 2000). A more specialized view of an avatar extends the simple image of a human being on a computer screen by enabling subjects to actually interact with their virtual environment, or even control it, and manipulate certain stimuli. However, only a few studies have actually implemented this approach in their experimental design since it lacks a necessary degree of standardized performance across subjects’ exposure to the VR environment [e.g., (Culbertson et al., 2010; Ferrer-García et al., 2010)]. Instead of a discrete distinction between avatars that allow for interactional behavior and those who do not, there are rather graduating steps in how an avatar is defined in individual studies, ranging from mere observation of virtual characters to actual manipulation of the environment using a virtual representation of the individual. Finding a common definition of an avatar in VR paradigms poses a challenge for recent psychiatric and neuroscientific research. For once, if standardized, it will set the framework for a deeper understanding of the socioenvironmental mechanisms of craving. Also, it eventually might lead to future advances in the treatment of SUDs. Hence, in this section, we refer to an avatar as a virtual representation of a person that *interacts* with the subject. This incorporates people in a virtual environment, which actively approach a subject (i.e., in order to offer them a cigarette) instead of performing passively observed substance-related behavior. In some studies, an avatar approached the user proposing to share an alcoholic beverage or cigarettes, but since the user did not have the possibility to answer and to change the unfolding course of events, we considered this as a non-interaction and the avatar as being a singular part of the environment.

The most frequent implementation of avatars in VR is as part of an environment closely related to specific substance use behavior. Due to standardized timing of stimuli exposure, researchers are capable to disentangle the effect of interaction an avatar engages subjects with from other surrounding and also potentially craving-inducing cues. Studies using this kind of avatars consistently report that social interactions, as much as substance-related objects, increase craving compared to neutral cues and baseline craving before exposure (Bordnick et al., 2004, 2005; Saladin et al., 2006; Carter et al., 2008; Cho et al., 2008; Lee et al., 2008; Ferrer-García et al., 2010; Garcìa-Rodrìguez et al., 2012, 2013). Although most of these study designs provide the opportunity to assess the effect of social interaction in a substance-related environment, the exact nature of interaction is seldom specified (Bordnick et al., 2005) and no explicit statistical comparisons are made between changes in subjective craving induced by the environment itself and social interaction. Thus, how far social interaction benefited the induction of craving by increasing ecological validity of the virtual environment of these studies remains unclear. Studies and results described below are summarized in Table 2.

The work of Bordnick et al. (2004, 2005, 2008, 2009) has to be highlighted as one of the first to assess craving induction with VR implementing artificial social interactions. Taken together, their results successfully point out that complex virtual cues can induce cravings in a broad range of substances among chronic substance users. Moreover, the results seem to demonstrate that social interactions directed toward drug use are as effective as paraphernalia cues to induce craving. Their pilot study in smokers demonstrated that immersion in a smoking-related virtual environment, including interaction with avatars offering cigarettes to the subject, significantly induced cravings for tobacco. Moreover, smoking-related social interactions with avatars provoked the highest craving score compared to smoking-related inanimate materials (Bordnick et al., 2004). Subsequent investigation of physiological reactivity with the same VR paradigm in non-deprived nicotine-dependent smokers and yielded that smoking-related virtual cues, including interaction with smoking avatars, significantly induced cravings for cigarettes and increased skin conductance response (Bordnick et al., 2005). Of interest, smoking-related inanimate cue and social interaction conditions induced the same level of craving, with no significant increase of craving directly ascribable to interaction with avatars. Other works have similarly featured the use of VR and interactions with avatars in TUD subjects. Carter et al. (2008) found that cigarette-related environments and interaction with smoking avatars elicited greater cravings than a neutral scene (Carter et al., 2008). Using eight different cigarette-related environments with avatars, Ferrer-García et al. (2010) showed that craving levels were significantly correlated to the sense of presence within the VR context. This result is particularly interesting in perspective of creating flexible virtual environments (Ferrer-García et al., 2010). Moreover, this emphasizes the importance of assessing the sense of presence in future studies, as the sensation of craving inducing the sense of presence may also be a possible phenomenon. In a similar fashion, Garcìa-Rodrìguez et al. (2012) have assessed the validity of seven different virtual environments, in which subjects could interact with avatars and objects, to induce nicotine cravings. This study showed that levels of cue-induced craving may be induced differentially, depending on particular situations successfully recreated in VR. However, the last three studies discussed (Carter et al., 2008; Ferrer-García et al., 2010; Garcìa-Rodrìguez et al., 2012) did not differentiate the impact of social interaction with levels of induced craving. Interestingly, Garcìa-Rodrìguez et al. (2013) also investigated the effect of smoking a virtual cigarette in a virtual pub allowing the interaction with avatars. The action of manipulating and “smoking” the virtual cigarette induced a significant increase in subjective cravings and heartbeat rate. The authors propose that simulating the action of smoking, in a virtual environment or in real life, may provoke cravings and relapse, acting as a CS.

A few studies have also assessed virtual interactions and craving in AUD. Bordnick et al. investigated the effect of their paradigm used in TUD (Bordnick et al., 2004, 2005) and AUD subjects (Bordnick et al., 2008). Subjects were exposed to neutral and alcohol consumption related virtual environments, including a bar, interacted with a virtual bartender and could order their favorite drink. Moreover, olfactive cues mimicked the scent of the specific drink, adding to the realism. Again, the alcohol-related cues and virtual interactions significantly induced cravings compared to neutral scenes. In other studies on AUD, social interaction with avatars has been used for a more tangible purpose that is quite distinct from casual interaction in everyday scenarios, such as a bar or a party. These studies make use of experimental settings, in which an avatar confronts subjects with social pressure, that is, using persuasive methods to engage the counterpart into substance use behavior. Since there is only a limited amount of studies assessing the feasibility of virtual social pressure situations to induce craving, findings so far are somewhat inconsistent. Although studies agree that the presence of an avatar urging subjects to drink alcohol can successfully increase their subjective craving levels, different views have been proposed regarding the context social pressure is deployed in. Cho et al. (2008) found that an avatar providing social pressure seems to increase craving independent of any substance-related cues. Subjective levels of craving were higher in the presence of an avatar, irrespective if the scenario was neutral or associated with alcohol use behavior (Cho et al., 2008). However, results by Lee et al. (2008) indicated that settings with substance-related cues might have differential effects on craving, which appear to be additionally driven by diagnosis of SUD (Lee et al., 2008). While social pressure significantly induced craving for healthy individuals in both a neutral and alcohol-related environment, craving levels of individuals diagnosed with SUD were already increased by the mere presence of substance-related cues. An additional avatar providing social pressure did not have any effect on changes in subjective craving levels.

Research on social aspects of craving induction has been conducted in other SUDs as well; however, available results are less numerous. Bordnick et al. (2009) investigated VR cue reactivity in cannabis smokers. Using the same paradigm as in their study on tobacco, subjects were immersed in four virtual environments (including two neutral rooms) with one cue room and one social interaction “party” room and subsequently filled VAS on marijuana cravings (Bordnick et al., 2009). In the party room, subjects could interact with avatars and even “use” marijuana with them, hear music playing, and smell the scent of marijuana. Results show that both the inanimate cue room and social interaction room significantly induced cravings compared to the two neutral rooms; however, there was no additional significant effect when comparing the inanimate cue room to the social interaction one. With respect to the idea of complex drug-related social interactions in craving induction, it has to be mentioned that olfactive and auditory stimuli were also present in the inanimate cue room, thus creating a more realistic drug-related scene. Finally, Saladin et al. (2006) have also demonstrated that VR cues and social interactions with avatars could significantly increase cravings for crack cocaine in dependent individuals. The virtual contexts in which cravings were highest implied social interactions with avatars, such as scenes where the subject interacted with a crack cocaine dealer or where other avatars were using crack cocaine (Saladin et al., 2006). Of further interest, scenes depicting aversive stimuli related to crack cocaine use (i.e., a police raid in the crack den) seemed to induce lower levels of craving, Moreover, although crack cocaine-related scenes induced craving, subjects’ mood (measured with a self-reported happiness scale) decreased and remained low for the remainder of the experiment. The authors propose that this decrease in mood might imply anticipated anxiety in the face of craving and withdrawal symptoms.

In summary, virtual interactions with avatars in different contexts can significantly induce cravings for multiple substances, with several results available on cigarette smoking and alcohol administration. Although avatars allow the presentation of a more complex and ecologically valid virtual environment, it is not clear if they induced cravings for substances in a manner different from other virtual settings. Real-life environments that are thought to induce cravings are usually populated with other people consuming substances or engaged in activities more or less related to drug use (i.e., dancing, listening to music, drinking, etc.). However, as already noted in the previous section on the use of virtual environments in cue-induced craving, the presence in the specific environment alone is sufficient to induce a significant increase in subjective cravings. Additional studies will, therefore, be needed to specifically investigate the difference between environmental and social cues in craving induction. The use of neurobiological correlates may prove to be essential in the hypothetical differentiation of such cues, as social interactions are more complex and engage different brain pathways than simple sensorial attention to present cues.

### Virtual reality as a beneficial addition to cue-exposure therapy in SUDs

Traditional CET designs in the treatment of SUD suffer from mixed findings regarding its efficacy in clinical populations (Conklin and Tiffanny, 2002; Kaplan et al., 2011). While there is experimental and meta-analytic evidence that exposure to VR cues might serve as an effective addition to CET approaches in anxiety disorders (Meyerbröker and Emmelkamp, 2010; Gonçalves et al., 2012), its beneficial effect with respect to SUDs still needs to be validated. VR CET in the context of SUD is assumed to induce states of craving by exposing individuals to environments closely related to everyday life and typical drug administration scenarios (Lee et al., 2004). This approach exceeds traditional CET designs, which mainly draw on substance-related cues detached from socioenvironmental or *in vivo* scenarios (Drummond and Glautier, 1994). However, since there are only few studies systematically examining the efficacy of VR in CET settings, overall findings to date are rather preliminary (Table 3). Studies within this line of research usually assess the feasibility of VR CET in a three-folded fashion. First, changes in subjective craving levels are determined after each session of exposure to either substance-related or neutral VR cues. Second, physiological responses, such as heart rate, skin conductance, or changes in cerebral blood flow by means of fMRI provide objective tools of craving assessment, which are independent of subjective ratings stated by the subjects. Third, an overall treatment effect is derived from comparing changes in subjective craving ratings and physiological responses before and after completion of the CET.

**Table 3:** **Summary of studies assessing the efficacy of VR in the context of cue-exposure therapy**.

Author (Year)	Type^a^	Abstinence^b^	VR Therapy^c^	No. of Sessions^d^	Session Spacing^e^	VR Cues^f^	Treatment effects on subjective craving levels^g^	Treatment effects on physiological correlates of craving^h^
**ALCOHOL**								
Lee et al. (2009)	CT	Y	CBT	10	nW	E	VR therapy led to a decrease in craving levels after 10 sessions when paired with aversive stimulation	Increase in EEG alpha-power at right frontal sites
**NICOTINE**								
Choi et al. (2011)	CT	N	CET	4	W	E	VR increased craving for smoking related compared to neutral cues; VR CET led to a decrease in craving levels after 4 weeks	SC and EMG increased during social situations compared to both smoking-related objects and neutral cues
Lee et al. (2004)	CT	N	CET	6		E, S	VR CET did not result in a decrease of craving after 6 sessions of treatment	
Moon and Lee (2009)	CT	N	CET	6		E	VR CET did not result in a decrease of craving after 6 sessions of treatment	Smoking-related VR cues increased activity in PFC and ACC compared to neutral cues; VR CET led to a decrease in PFC activity.
Park et al. (2014)	CT	N	CET	4	W	E, S	VR CET did not result in a decrease of craving after 4 sessions of treatment	CO-levels decreased and remained stable until the end of the treatment
Pericot-Valverde et al. (2012)	CR	N	CET	6	W	E	Decrease in subjective reports of craving during the course of the treatment	CO-levels decreased and remained stable until the end of the treatment

*^a^CT, clinical trial; CR, case report*.

*^b^N, no abstinence at the beginning of the treatment; Y, abstinence at the beginning of the treatment*.

*^c^CET, cue-exposure therapy; CBT, cognitive-behavioral therapy*.

*^d^Number of total treatment sessions*.

*^e^Treatment sessions took place either once (W) or twice (nW) a week*.

*^f^E, virtual environments; S, virtual social interaction*.

*^g^VR CET, virtual reality cue-exposure therapy*.

*^h^EEG, electroencephalogram; SC, skin conductance reactivity; EMG, electromyogram; PFC, pre-frontal cortex; ACC, anterior cingulate cortex; CO, breath carbon monoxide*.

The majority of studies addressing the efficacy of VR CET in a therapeutic context assessed populations with a diagnosis of nicotine dependence. One of the first studies to investigate the use of VR CET in cigarette smokers was performed by Lee et al. (2004). Encouraged by their previous findings that VR environments induced higher levels of subjective craving ratings compared to classical pictorial cues (Lee et al., 2003), the group implemented a VR paradigm into a CET setting with current male smokers (Lee et al., 2004). Subjects were exposed to VR environments and social situations and assessed with respect to subjective craving, as well as number of daily smoked cigarettes. Although results indicated a trend toward decreasing numbers of smoked cigarettes from pre- to post-treatment, neither this outcome nor subjective craving ratings reached statistical significance during or at the end of the VR CET. Given that Lee et al. (2004) conducted a preliminary study, a few issues might explain the lack of treatment effects on smoking urges and actual number of smoked cigarettes. Of note, the sample in this study could retrospectively be classified as non-clinical since no differentiation was made between frequent smokers and those qualifying for diagnostic criteria of nicotine dependence. Moreover, there was no methodological assessment of differences between the effects of VR in inducing craving compared to traditional pictorial or neutral cues. This comparison is necessary to argue toward a statistically and clinically meaningful treatment effect, which can be specifically attributed to exposure to VR cues.

Indeed, systematical subsequent research by Choi et al. (2011) provided evidence that exposure to VR settings successfully induced cigarette craving in TUD subjects (Choi et al., 2011). Increases in craving levels were observed by means of both higher ratings of subjective craving as well as elevated physiological responses [i.e., increased levels of skin conductance and electromyography (EMG)] to substance-related and social cues when compared to neutral control scenarios. These findings are in line with previous studies (Bordnick et al., 2004, 2005; Baumann and Sayette, 2006; Garcìa-Rodrìguez et al., 2012, 2013; Acker and MacKillop, 2013) showing that substance-related objects and social scenarios induce craving in individuals diagnosed with SUD. Moreover, subjects showed increased physiological responses to social rather than substance-related cues in virtual environments, although no differences were found in terms of subjective craving. Even more important, subjects’ cue reactivity to smoking-related stimuli decreased from the first to last session of CET subjectively and physiologically, hinting toward a general effect of VR CET in reduction of craving levels.

However, Choi et al. (2011) refrained from including a group of healthy subjects exposed to the same experimental conditions, or a group of TUD subjects receiving conventional CET in their study design. It remains, therefore, questionable how far the presented results can be viewed in favor of VR as a therapeutic aid in CET. This notion is supported by findings on the effect of VR CET in a previously conducted study (Moon and Lee, 2009). Here, men with TUD were exposed to substance-related and neutral VR scenarios in an fMRI scanner. Smoking-related VR cues (i.e., a virtual bar with paraphernalia or smoking avatars at a party) increased activity in the PFC and ACC compared to neutral cues providing insight into functionally higher cognitive cortical areas, which are involved in cognitive control and, thus, potential drug-seeking behavior (Feil et al., 2010). Additionally, VR CET over a total of six sessions led to a decrease in PFC activity from the first to the last session. However, Moon and Lee (2009) did not find any effect of VR CET on subjective craving levels after six sessions of treatment. The non-clinical population that was used in this particular study might again explain the lack of statistical significance in subjects’ differential behavioral responses to substance-related and neutral cues. Hence, results of this study need again to be interpreted with care.

Although studies assessing the efficacy of VR CET are highly ambiguous, their results might provide a framework for individualized treatment approaches. A recent case report by Pericot-Valverde et al. (2012) gives insight into how an elaborate treatment plan might implement VR scenarios into CET in a personalized fashion. In this study, a woman successfully quit smoking by being administered to an extensive individualized treatment plan that included both relapse training and VR CET over a course of 6 weeks (Pericot-Valverde et al., 2012). Prior to any treatment, the subject was asked to identify VR cues that might specifically induce an urge to smoke. These cues were then implemented into a VR environment. Subjective ratings of craving, as well as breath carbon monoxide levels and number of smoked cigarettes decreased over time during treatment. Since this example is a case report and there was no follow-up assessment to determine potential relapse after completion of the treatment program, it might be considered a first step in optimizing treatment designs that make use of VR CET.

Studies reviewed here provide insight into possible treatment aids of VR CET. However, little effort has been made so far to compare VR CET to other treatment approaches. A recently published study by Park et al. (2014) compared VR CET to conventional cognitive behavioral therapy (CBT) in treatment-seeking male smokers. Subjects underwent either a treatment plan, which involved VR CET – including exposure to substance-related environments and social interactions – or CBT (Park et al., 2014). Both VR CET and CBT significantly reduced numbers of smoked cigarettes and levels of breath carbon monoxide from pre- to post-treatment and even weeks after completion of the program. However, neither individuals assigned to VR CET nor those in the CBT group experienced decreases in subjective craving levels. Additionally, since there were no apparent differences between VR CET and CBT on all treatment outcomes, assignment to VR CET appeared to produce a treatment effect that could be at least considered similar to traditional CBT.

While studies on individuals with a TUD diagnosis are growing in number, there is only limited literature regarding the use of VR in clinical studies assessing behavioral treatment approaches in alcohol-dependent populations. Lee et al. (2009) examined the use of a VR therapy approach, in male individuals with an AUD diagnosis and healthy controls while recording changes in EEG alpha frequency band power of fronto-parietal cortical areas (Lee et al., 2009). The paradigm of this study differs from CET in a way that it does not follow the approach of Pavlovian CS-CR extinction, but adds an aversive stimulus to the model of classical conditioning, usually referred to as *aversion therapy*. Individuals undergo a variety of scenarios, which set them in certain affective states including relaxation, as well as positive and negative arousal. The combination of positive and negative affective states (i.e., craving a specific drug and immediate subsequent multisensory aversive stimulation, such as showing a person vomiting and simulating the smell and taste of vomit) leads to a restructuring of the CS-response relation in addiction and should eventually result in avoidance of the administered drug. Despite different approaches in the actual treatment of addiction in CET and aversion therapy, the implementation of VR scenarios serves the same purpose in both cases, that is, exposing individuals to substance-related cues that result in an increase in craving levels. Hence, in the study by Lee et al. (2009), exposure to virtual substance-related objects and environments led to a larger increase in craving levels for alcohol-dependent subjects compared to healthy controls. Additionally, a decrease in subjective craving levels as well as increases in fronto-parietal EEG alpha-power after 10 sessions of therapy was more prominent for VR therapy when compared to aversion therapy, indicating that the addition of VR scenarios to CBT related paradigms might result in improved treatment success.

Accumulated results of studies reviewed here point toward a decrease in the amount of substance intake per day during the course of VR CET (Lee et al., 2004; Choi et al., 2011; Pericot-Valverde et al., 2012; Park et al., 2014). With respect to its therapeutic goal, that is, reducing or stopping the administration of drugs, VR CET holds promising results at least in the short term. To validate its feasibility as a treatment for prospective abstinence in SUDs, follow-up assessments need to take place, which were only performed in one of the studies reviewed here (Park et al., 2014). This notion is crucial taking into account that especially craving for substances can occur after longer periods of abstinence leading to potential relapse (Paliwal et al., 2008; Galloway and Singleton, 2009). Additionally, only little inference can be made in how far a treatment effect can be attributed to the use of VR environments and social interactions during exposure to substance-related cues. Contrary to findings on treatment effects regarding substance intake, VR CET did not affect levels of subjective craving in most studies reviewed here. This might be due to heterogeneity in study designs, which makes it difficult to draw an overall picture on the putative superior effect of substance-related VR cues over neutral VR or traditional substance-related cues to induce craving for drugs. Only a few studies distinguished between craving induction during cue exposure and subsequent decrease in subjective craving, amount of substance intake, and physiological correlates of craving (Choi et al., 2011). Moreover, most studies do not report the former by comparing substance-related to neutral VR or traditional cues, such as pictures, and mostly relied on previous work, which is substance specific still fairly remote in number (see Tables 1 and 2). The delineation of successful craving induction and its reduction during the course of a treatment is of utmost importance to attribute a benefit of treatment aid to the use of VR cues. This will eventually ensure that reduction in substance intake is not mainly a result of treatment irrespective of the type of exposure, which in the long run might again lead to potential craving and relapse.

Findings by Lee et al. (2009) suggest that a decrease in subjective craving levels is achieved by means of combined VR cue exposure and aversion therapy. Hence, the concept of classical conditioning as the fundamental basis of persistent drug use and abuse might not be a sufficient theoretical framework with respect to the treatment of SUDs. Although extinction of acquired reactivity toward substance-related cues appears quite straightforward, its application in the context of SUD treatment might be yet fragmentary. This is in line with previous findings in both animal models (Conklin and Tiffanny, 2002) and human studies (Kaplan et al., 2011) challenging the efficacy of current CET as a stand-alone treatment approach in SUD. An emotional component inherent to most substance-related cues exceeds the idea of a simple classical Pavlovian learning paradigm and reflects affective states conceivably refractory to re-conditioning approaches (Havermans and Jansen, 2003).

Besides theoretical considerations, treatment success often strongly depends on factors such as sex, age, or duration of treatment (Zilberman et al., 2003; Kaplan et al., 2011; Koechl et al., 2012). Interestingly, one VR CET case report revealed a decrease in subjective craving levels for nicotine (Pericot-Valverde et al., 2012), whereas studies assessing samples of individuals mainly did not yield corresponding results. This highlights the potential of individualized CET plans for successful treatment of SUD in both stopping current substance abuse and avoiding future relapse. In light of a continuously growing, yet methodologically inconsistent, number of findings regarding VR CET in SUD on a group level, personalized treatment can serve as a key in overcoming practical and statistical issues that might circumvent therapeutic efficacy in individuals. Individualized treatment in SUD has previously been suggested as a promising approach to manage individual variability in AUD patients (Mann and Hermann, 2010). Additionally, cue reactivity, and thus craving, is associated with lower levels of substance dependency (Watson et al., 2010) and rates of relapse (Conklin et al., 2012). In context of traditional CET, cue reactivity is reflected by changes in activation of the mesolimbic circuit (Vollstädt-Klein et al., 2011). Vollstädt-Klein et al. (2011) observed a decrease of activation within areas including the ACC, PFC, and ventral striatum after CET. With respect to findings that linked increases in ACC and PFC to higher levels of craving (Moon and Lee, 2009), one could argue that VR CET might have the properties to downregulate hyper-activation of dopaminergic mesolimbic areas. Hence, increasing ecological cue validity in virtual settings might subsequently result in elevated cue reactivity and respective changes in mesolimbic activity, facilitating VR CET efficacy on at least the individual level.

## General Discussion

In sum, immersing subjects in substance-related virtual environments can successfully induce craving for different substances. Substance-related VR cues compared to neutral cues induced significant craving in several studies. Moreover, studies implementing social interactions with avatars in cue-provoked paradigm also reported increases in craving compared to neutral VR immersion. It thus seems that VR offers a good technical alternative to standard videos and photographs in standard cue-provoked paradigms and that VR can successfully integrate a simulation of social interaction in such paradigms. We believe this is of great importance as social interactions are often reported as craving agents and incentives to consume. However, the difference in craving induction between the immersion in a virtual environment and the interaction with avatars remains to be tested.

Virtual reality thus shows an interesting potential in the improvement of actual assessment of craving in SUDs. VR is a flexible and controllable tool in the sense that cues can easily be modified to certain patient’s requirements or condition. This specificity of environments and avatars thus holds great promise for the development of individualized care and treatment in SUDs. Thus, VR may help reach a significantly higher level of ecological validity. Although traditional craving paradigms use validated sets of photographs and videos, VR directly involves the subject in simulated situations and this may significantly improve craving induction and, further down the line, sensitization behavioral therapy. Moreover, since SUDs are complex conditions evolving on several weeks and months and comprising various stages, VR may be used in these different stages i.e., before the appearance of craving. Although it offers limited support in the study of actual substance consumption, VR may help understand other stages in the development cycle of substance use. One of the key concepts in this line of discussion is *sense of presence*, which describes individual experiences of immersion, naturalism, and realism, or simply the “being there” in a virtual environment (Lombard et al., 2009). Hence, when assessed systematically, sense of presence can serve as an indicator in how far VR settings resemble real-life situations. Rigorously assessing sense of presence and immersion will thus be crucial in future works on SUDs with VR. While increased sense of presence can be linked to elevated individual engagement in VR, it also might result in negative effects, including sensations of vertigo or nausea during or after VR exposure (De Leo et al., 2014). These adverse physiological reactions are generally referred to as *cybersickness*. Cybersickness is mainly attributed to conflicts in sensory processing in artificial virtual environments and is, thus, closely related to the concept of sense of presence (So et al., 2001; Kiryu and So, 2007). Although a standardized tool for assessment of cybersickness is already available for a longer period of time [Simulator Sickness Questionnaire (SSQ); (Kennedy et al., 1993)], only a minority of studies reviewed here reported its use. Scores of cybersickness questionnaires were generally low and indicate that it did not affect behavioral or physiological outcome measures (Lee et al., 2004, 2005; Choi et al., 2011). However, one study (Baumann and Sayette, 2006) reported a single case of severe cybersickness that led to incomplete testing and subsequent dropout from statistical analysis. Hence, the issue of cybersickness might impact on both individual health and the validity of study outcomes. Finally, although it requires an important level of expertise to program and set-up VR paradigms, it is cost-effective, easily combinable with pharmaco- and psychotherapies, and presents few side effects. A recent review (Bush, 2008) points out the fact that VR may be more desirable in several clinical settings, as it is more private and less embarrassing for patients than *in vivo* behavioral therapy.

The interesting potential of VR in behavioral research and medicine may also be grasped in different contexts. For example, compulsive overeating (Hone-Blanchet and Fecteau, 2014) and gambling (Leeman and Potenza, 2011) are often compared to SUDs because of their compulsive component and appearance of craving. A few studies have investigated the use of VR to provoke food cravings (Ferrer-García et al., 2013; Ledoux et al., 2013). Moreover, researchers propose that VR may help in the assessment and treatment of obesity (Bordnick et al., 2011). Thus, VR holds promising potential in developing applications in neurosciences and may help promoting individualized care in future clinical psychology.

## Conflict of Interest Statement

The authors declare that the research was conducted in the absence of any commercial or financial relationships that could be construed as a potential conflict of interest.
